# Seismology-based early identification of dam-formation landquake events

**DOI:** 10.1038/srep19259

**Published:** 2016-01-12

**Authors:** Wei-An Chao, Li Zhao, Su-Chin Chen, Yih-Min Wu, Chi-Hsuan Chen, Hsin-Hua Huang

**Affiliations:** 1 Department of Geosciences, National Taiwan University, Taipei 10617, Taiwan; 2 Institute of Earth Sciences, Academia Sinica, Nankang, Taipei 11529, Taiwan; 3 Department of Soil and Water Conservation, National Chung Hsing University, Taichung 40227, Taiwan; 4 National Center for Research on Earthquake Engineering, National Applied Research Laboratories, Taipei 10668, Taiwan; 5 Central Geological Survey, MOEA, Taipei 23568, Taiwan; 6 Department of Geology and Geophysics, University of Utah, Salt Lake City, USA; 7 Seismological Laboratory, California Institute of Technology, Pasadena, CA 91125, USA

## Abstract

Flooding resulting from the bursting of dams formed by landquake events such as rock avalanches, landslides and debris flows can lead to serious bank erosion and inundation of populated areas near rivers. Seismic waves can be generated by landquake events which can be described as time-dependent forces (unloading/reloading cycles) acting on the Earth. In this study, we conduct inversions of long-period (LP, period ≥20 s) waveforms for the landquake force histories (LFHs) of ten events, which provide quantitative characterization of the initiation, propagation and termination stages of the slope failures. When the results obtained from LP waveforms are analyzed together with high-frequency (HF, 1–3 Hz) seismic signals, we find a relatively strong late-arriving seismic phase (dubbed Dam-forming phase or *D*-phase) recorded clearly in the HF waveforms at the closest stations, which potentially marks the time when the collapsed masses sliding into river and perhaps even impacting the topographic barrier on the opposite bank. Consequently, our approach to analyzing the LP and HF waveforms developed in this study has a high potential for identifying five dam-forming landquake events (DFLEs) in near real-time using broadband seismic records, which can provide timely warnings of the impending floods to downstream residents.

The formation of a landquake dam results from the natural blockage of the river channels by mass wasting from hillslopes. The existence time of a landquake dam directly determines the seriousness of the subsequent dam breaching. In the global catalog of landquake dam failures, 27% of the landquake dams failed within a day after formation^1^. Due to the insufficient reaction time, the failure of a landquake dam often results in loss of lives. For example, a catastrophic Shiaolin landquake occurred during the passage of the 2009 Typhoon Morakot across Taiwan, which resulted in 465 deaths in Shiaolin Village and the short-lived (~104 minutes) landquake dam blocking the Chishan River^2^. For potentially large landquake dams, early identification of DFLEs is essential to organizing appropriate preventive actions in due time.

Seismic sources can be described in the point-source approximation by a symmetric second-order moment tensor (MT) that can be decomposed into pure double-couple (DC), compensated linear vector dipole (CLVD) and isotropic (ISO) components^3^,^4^. However, source mechanisms of non-fault earthquakes (i.e. landquakes) cannot be parameterized by a moment tensor (MT). In the past decade, force time functions of sinusoidal shape have been widely used to study Earth surface processes such as glacial earthquakes^5^,^6^ and landquakes^7^,^8^,^9^,^10^. Long-period data have been used to deduce the forces on the ground surface with positive and negative peaks related to the acceleration and deceleration stages of the block mass from initial destabilization to its arrest. In contrast, analysis of the short-period (≥1 Hz) data provides a more complete understanding on the dynamics of the slope failures^11^,^12^,^13^. Here we propose a new approach based on analyzing HF and LP waveforms to providing rapid warning when a large landquake is flowing/sliding towards a river, possibly leading to a dam that may subsequently breach and generate destructive flooding downstream.

## Results

### Inversions of LP waveforms

**Figure 1:**
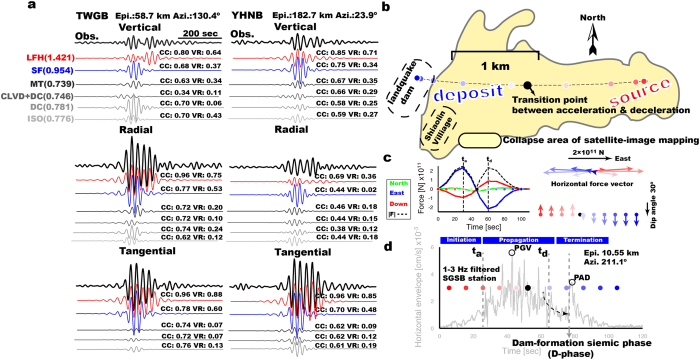
General source inversion (GSI) and landquake dynamics for Shiaolin event. (**a**) Examples of fits at two stations between records (black) and synthetic seismograms calculated for different source mechanisms including landquake force history (LFH, red), single force (SF, blue), and full moment tensor (MT) and its deviatoric moment tensor (CLVD + DC), double couple (DC), and isotropic (ISO) components plotted with different gray levels. All waveforms are filtered to 0.025–0.05 Hz, and the fitness values are typically between 0.746 and 1.421. The normalized cross-correlation coefficient (CC) and variance reduction (VR) are given at the end of each synthetic trace. The station name, epicentral distance, and station azimuth are given at the top. (**b**) Inferred collapsed-mass trajectory. The colored region shows the collapse area of the Shiaolin event mapped by the Central Geological Survey of Taiwan. Color dots indicate the locations of the center of collapsed-mass along run-out path trajectory. The black dot shows the transition spot from acceleration to deceleration. Maps are created using GMT (Generic Mapping Tools, http://gmt.soest.hawaii.edu/) software. (**c**) LFH of each component (green: north; blue: east; red: down) and the absolute value of the force vector (dashed line). Time-dependent force vectors acting on the Earth are shown in the right panel. (**d**) Filtered horizontal envelope functions recorded at the closest Station SGSB. Open circles depict the peak ground velocity (PGV) and peak amplitude of *D*-phase (PAD). The dashed arrow shows the smooth decay of short-period seismic signals. All color dots correspond to those in (**b**) of the same color during the time progression from 0 to 100 s in the LFH result. The vertical dashed lines indicate the onset of maximum acceleration (*t*_a_), maximum deceleration (*t*_d_), and dam-forming phase (*D*-phase), respectively.

Successful waveform modeling requires good knowledge about the source location. For events studied here, published results^13^,^14^ for the locations of landquakes are available. We also carried out an automated detection of the earthquake and/or landquake activity using detection algorithm developed in our previous studies^13^,^15^ and then performed a systematic time-frequency analysis to manually identify landquake events with near triangular-shaped spectrograms. Finally, we applied a cross-correlation technique^13^ to determine the locations of newly detected events. Based on a general source inversion (GSI) approach (see section Methods), we first conduct inversions of the LP waveforms from broadband seismic stations for source mechanisms (including single-force (SF), DC, CLVD and ISO components). Modeling results show that synthetics for a SF mechanism fit the observed seismograms generated by the Shiaolin landquake event best, with a fitness of 0.954 (Fig. 1a). For an aim of automatically identifying landquake source, comparison with waveform fits by tectonic source mechanisms (fitness ≤0.781) demonstrates that in practice landquake source can be simply identified by observing the improvement in waveform fitness values (Fig. 1a) without the manual identification of triangular-shape spectrogram in the procedure of event detection. Aforementioned automatic identification is workable for all landquake events. Figure S1 shows the examples of resulting GSI for two DFLEs and three events without dam-formations. Second, we further inverted the LP seismic data to obtain the landquake force history (LFH) exerted on the Earth by the moving block mass (see section Methods). For these inversions, the modeled waveforms fit the observed seismograms with fitness values typically between 0.791 and 1.596, and the maximum force *F*_max_ spans the range between 0.3 and 61.6 × 10^10^ N (Table S1). Because the LFH approach utilizes full waveforms from each component (north, east and vertical), it can be done with only a few stations with good signal-to-noise ratio (SNR) and well azimuth coverage. In case of the Event ID Taimali#2, there are only two stations with good SNR that can be used in waveform modeling, which are sufficient for reaching a reliable solution (Table S1 and Figure S2). The resulting LFH provides three-dimensional (3-D) force vectors along a sliding slope that is crucial for understanding landquake dynamics. Assuming a relatively constant block mass, the three-component acceleration time series of the moving block can be obtained by dividing the LFH results by the mass. A subsequent double integration of the acceleration yields the displacement corresponding to the run-out path of the landquake event. However, there is a trade-off between mass and displacement in the aforementioned calculation. In this study, the block mass of the landquake is obtained through a grid-search scheme by fitting the observed run-out distances identified in remote-sensing images^13^,^14^. In the case of the Shiaolin landquake event, we estimated the vertical and horizontal total block-mass displacements (*D*_v_ and *D*_h_) of 1047 m and 2624 m, respectively, which leads to a mass of m = 8 × 10^10 ^kg (Table S1) and the trajectory shown in Fig. 1b. Assuming an average density of 2500 kg/m^3^, the estimated collapse volume is ~32 million m^3^. These estimated values are broadly consistent with field measurements and image mapping results^2^.

### Dynamic landquake processes and identification of DFLEs

The estimated LFH exhibits a roughly sinusoidal shape with a duration of ~100 s (Fig. 1c). In the first 47 s the force vectors point consistently to the east with an upward vertical component (red dots and arrows in Fig. 1c), indicating a reaction to the acceleration of the moving block mass downhill to the west (red dots in Fig. 1b). The force vectors induced by the deceleration reveal a westward direction, in the same direction of the sliding mass (blue dots in Fig. 1b,c and blue arrows in Fig. 1c). Previous works have shown that the seismic signals excited by landquakes are dominated by Rayleigh surface waves and/or S waves^12^,^13^,^16^. Figure S3 clearly shows the peak HF envelope amplitude, which would be associated with the greatest mass impact on the ground, with a propagation velocity of ~2.8 km/s close to the *S*-wave velocity. In order to compare the LFH results with the HF seismic signals, we calculate travel time from the corresponding point source on the resulting trajectory (color dots shown in Fig. 1b) to the closest station. For landquake events occurred in the mountain area of Taiwan, we perform a 3-D ray tracing^17^ through the shear-speed model^18^, which minimizes the effect of lateral heterogeneities on travel time predictions in LFH responses from the point sources to the closest station. For the Oso-steelhead event, a regional 1-D layered model^19^ was used for travel time calculation. The interpretation that follows is insensitive to small discrepancies in the used velocity model. To extract the HF seismic signals recorded at the closest station, we calculate the root-mean-square amplitudes of the filtered (1–3 Hz) horizontal-component waveforms to obtain the horizontal envelope functions. Furthermore, a combined analysis of the HF horizontal envelope function with dynamics inferred from LP waveform modeling provides a quantitative characterization of the initiation, propagation and termination stages of the landquake event. At the closest station SGSB for the Shiaolin landquake event (Figure S4a), relatively small and low-frequency seismic amplitudes are generated during the initiation stage (before time *t*_a_ in Fig. 1d) by both the slow mass movement and failure-slope detachment. Then, higher-frequency amplitudes, which might relate to grain impacts and complex process of block mass movement^12^,^13^, are visible in the propagation stage. After the maximum deceleration time (*t*_d_ in Fig. 1d), seismic signals rapidly decay, suggesting that the block mass is no longer moving. However, a strong late-arriving seismic phase (named Dam-forming phase or *D*-phase) in the HF envelope function can be observed during the termination stage (Fig. 1d). Comparing the timing of *D*-phase with the corresponding position of the block mass along its trajectory shows that the mass has reached the river channel (Fig. 1b). Thus, this *D*-phase is likely generated by the collapsed-mass sliding into the river and blocking it, and perhaps even further impacting the opposite river bank. Recent studies noted a similar short-period seismic energy appearing suddenly when the sliding mass reaches a topographic barrier^9^,^10^. In this study, our aim is to detect the *D*-phase in the closest seismic records, which can help identify the DFLEs.

**Figure 2:**
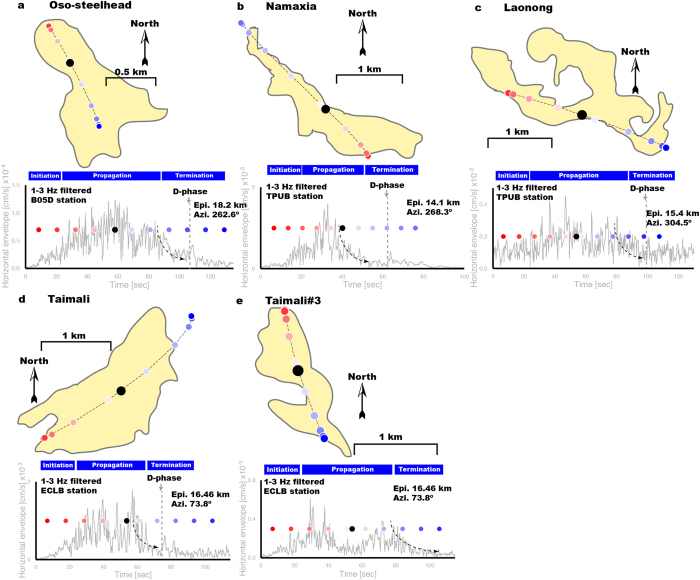
Timing of *D*-phase and the corresponding position of the moving block mass. Results of seismologically determined trajectory and SP envelope functions for four DFLEs: (**a**) Oso-steelhead, (**b**) Namaxia, (**c**) Laonong and (**d**) Taimali, and for an event without dam-formation: (**e**) Taimali#3. Station azimuth and epicentral distance are given in the top-right corner of each envelope function. Color dots indicate the locations of the center of collapsed-mass along run-out path trajectory. Black dot shows the transition point from acceleration to deceleration. All colored dots represent the time progression in the LFH result. The color region in each plot shows the collapse area from published satellite-image mapping results^13^,^14^. Maps are created using GMT (Generic Mapping Tools, http://gmt.soest.hawaii.edu/) software.

We applied our approach to a total of ten events, comprising five DFLEs (Event ID Shiaolin, Taimali, Laonong, Namaxia, and Oso-steelhead in Table S1), which were well recorded by regional broadband seismic networks (Figure S4 and see section Methods). Results of the estimated trajectory and HF envelope functions for four DFLEs and a non-dam-forming are shown in Fig. 2. Clear *D*-phase signals with bursts of HF radiations consistently appear in the termination stage of DFLEs. However, the relatively weak amplitude of *D*-phase signal can be contaminated by the large noise signals. Figure S3 shows the HF horizontal envelope functions recorded at BATS stations with a wide range of epicentral distances for DFLEs of Shiaolin and Taimali. Indeed, *D*-phase signals in the horizontal envelope functions can be observed only on closer stations (source-to-receiver distance ≤40 km). A possible solution to enhance the SNR of *D*-phase is to use waveform-stacking method that can improve the reliability of the *D*-phase detection. Therefore, amplitudes in these *D*-phase signals may be influenced by topographic changes of the river channel and the momentum of the mass sliding into the river. Even more notably, two landquake events (Events Taimali and Taimali#3 in Table S1) recorded by several broadband stations are only two minutes apart in HF envelope functions (Figure S5). Distance between two landquake events is about 2 km (Table S1). Our previous work^13^ based on HF waveforms has shown that the distance between the mean locations of the associated landquakes determined by satellite-image mapping and seismological means is ~2 km. Thus, for correctly identifying and correlating the events with mapped collapse area, which occur closely in space and time, relying on HF seismic signals only is a very challenging problem. Here, modeling of LP waveforms provides crucial information of landquake dynamics (e.g., trajectory) for identifying events occurred in the vicinity (Fig. 2d,e and Table S1). Resulting trajectories of Events Taimali and Taimali#3 show the northwest and southward directions, respectively. For landquake Taimali#3, its trajectory is limited in failure slope and do not extend to the riverside. Consequently, there is no *D*-phase signal in its HF envelope functions (Fig. 2e).

### Collapsed mass versus landquake force and magnitude

**Figure 3:**
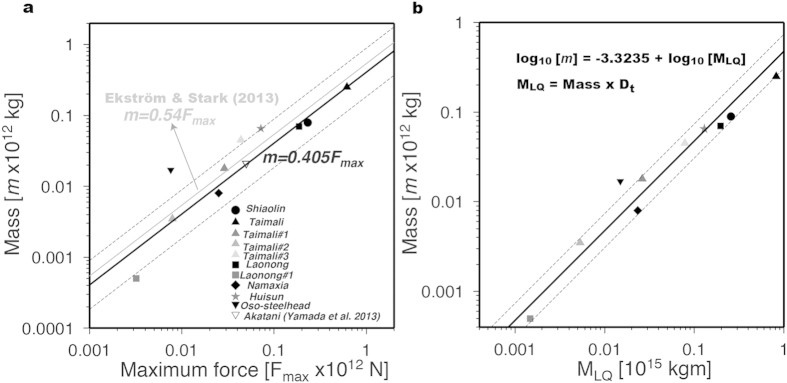
Regression scaling relations. Estimated mass (*m*) of sliding block versus (**a**) maximum force *F*_max_, and (**b**) magnitude of landquake event *M*_LQ_. The black solid lines show regression lines and the two dashed lines indicate the range of two standard deviation. Symbols of different gray levels indicated different events listed in Extended Data Table 1. Gray solid line in (**a**) is the regression result of Ekström & Stark^8^. Five DFLEs are depicted in solid black symbols.

Based on the aforementioned estimated values, we derive an empirical linear relationship between the estimated collapsed mass (*m*) and maximum force (*F*_max_). The results indicate a good linear relationship (*m* = 0.405*F*_max_, Fig. 3a). Nevertheless, a small discrepancy can be seen comparing with results derived from twenty-nine catastrophic events using global seismic data (ref. 8; gray line in Fig. 3a). Several factors may contribute to this discrepancy. In general, different source processes are responsible for the radiations of short- and long-period waves. Moreover, the spatial-temporal resolution of LFHs can be influenced by different frequency bands used in LP waveform modeling. In this study, we have used relatively higher frequency band for the LP signals (0.025–0.05 Hz; see section Methods), which is likely to be weak on the Global Seismographic Network due to attenuation. In particular, result from a previous study^9^ using seismic records of higher and broader frequency band (0.01–0.1 Hz) in tracing the dynamics of landquake event follows our relationship (open inverted triangle in Fig. 3a). Most notably, Event Oso-steelhead has an anomalous relationship between *m* and *F*_max_, which may be attributable to its geometrical characteristics since this event originated on a gentle slope (<20°) from an elevation of approximately 180 m, yet it still traveled a run-out distance of ~1 km (ref. 14). The integral of the moment rate yields the total moment which represents the size of earthquake^4^. Previous studies have proposed that the amplitude of twice-time-integrated force indicates the overall size of the landquake (ref. 20; *M*_LQ_ = *m* × *D*_t_, where 
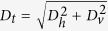
 is the travel distance along the failure slope). We further establish a linear relationship between mass (*m*) and landquake magnitude (*M*_LQ_). The result of the regression, as shown in Fig. 3b, has a pattern consistent with the scaling result in Fig. 3a. These relationships allow for a rapid determination of the collapse mass after the occurrence of a landquake, thus the trajectory of the sliding block can be calculated directly from the LFH results.

## Discussion

Our results demonstrate that seismic monitoring is a valuable tool for determining landquake source parameters and identifying DFLEs. However, our approach can only be used to study large and rapid landquake sources that generate LP seismic waves used in waveform modeling with the block model approximation, which may not be appropriate for two events with relatively small fitness (Events Laonong#1 and Namaxia in Table S1). In Taiwan, real-time moment tensor monitoring system (RMT, http://rmt.earth.sinica.edu.tw/) has been developed to provide information of earthquake source parameters in about two minutes after the occurrence of an earthquake, including the event origin time, hypocentral location, moment magnitude and focal mechanism^21^. In practical applications to landquakes, we can vastly expand the existing real-time broadband seismic networks in Taiwan to provide near-real-time landquake source mechanisms as part of routine operations. This is an important feature for the purpose of alerting relevant entities to the occurrence, location and magnitude of a catastrophic landquake event. Ideally, once the real-time seismograms reach a number of seismic stations, our approach merely takes a few seconds on a desktop computer to perform the LP waveform modeling. The total amount of computational time is proportional to the number of trial landquake sources, a scenario suitable for parallelization.

**Figure 4:**
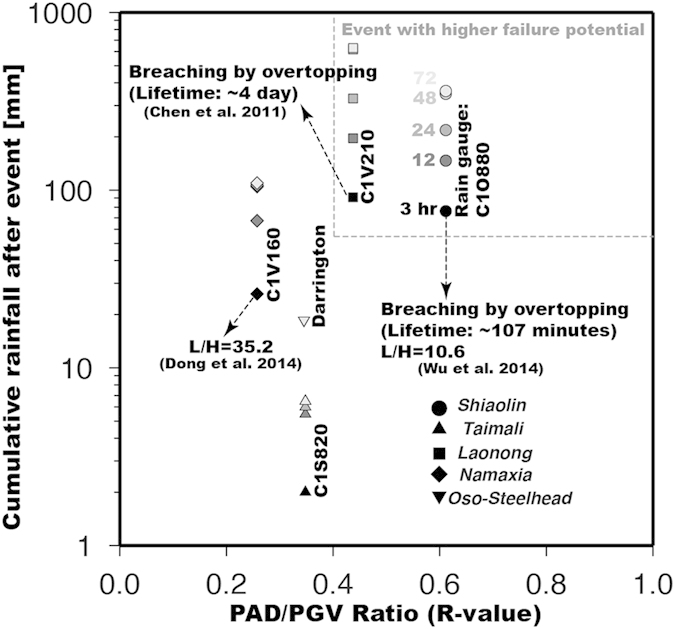
Identification of high-potential failure DFLEs. Different symbols correspond to different DFLEs listed in Table S1. Gray scale indicates the cumulative rainfall after the event for different time-periods. Names of rain gauge stations used in this study are given to the right of each symbols. L/H values are collected from published results^2^,^30^. The dashed line delineates regions of high-potential failure DFLEs.

The life span of a landquake dam depends on the stream hydrodynamics, geomorphologic factors, and the geometry and composition of the dam^1^. Previous works indicate that longer living dams have relatively high length-to-height ratio (ref. 22; L/H > 20). Here we propose a new parameter which is the ratio between the peak ground velocity (PGV) and the peak amplitude of *D*-phase (PAD) (Fig. 1e). The PGV value, which may be associated with the greatest mass impact on the failure slope^13^,^23^, is estimated from the HF horizontal envelope function. The PAD value can be related to the momentum release of dam formation. We can expect that a high PAD/PGV ratio (*R*-value) corresponds to a small L/H value. Thus, incorporating *R*-value into the meteorological data^14^,^24^ would therefore allow the early identification of DFLE with higher failure potential. Indeed, the short-lived (~104 minutes) Shiaolin landquake dam can be quickly identified by a relatively high *R*-value and a low L/H value, coinciding with the most intense and prolonged rainfall (Fig. 4). Our proposed approach to the combined analysis of LP and HF seismic signals is very effective for a rapid determination of the source dynamics and for identifying DFLEs (Fig. 1). It facilitates real-time landquake monitoring and downstream early warning systems, which provide important, useful and timely information for mitigating landquake and dam-breach hazards.

## Methods

### Data

Records used for nine landquake events in Taiwan were provided by the Broadband Array in Taiwan for Seismology (BATS, http://bats.earth.sinica.edu.tw/) data center. Broadband records from TA and UW seismic networks (IRIS Data Management Center, http://dx.doi.org/doi:10.7914/SN/II) were used for the 2014 Oso-steelhead landquake event occurred in Washington, USA. Seismic data processing involves deconvolving instrument responses, integrating from ground velocity to displacement, rotating the horizontal components to radial and transverse directions for each station, and an application of fourth-order minimum-phase Butterworth band-pass filter with periods between 0.025 Hz and 0.05 Hz. Different weightings are assigned in the inversion according to the quality (signal-to-noise ratio, SNR) of the filtered waveforms.

### General Source Inversion (GSI)

A more flexible approach to full-waveform inversion is developed in this study, which models the seismic source as a full moment tensor (MT) plus a single-force (SF). We follow the inversion algorithm of Kikuchi & Kanamori^25^, which decomposes the MT into five double couples (DCs) and an isotropic (ISO) source. The full MT is then represented by a linear combination of six elementary moment tensors. A SF consists of three orthogonal (north, east and vertical) forces. Thus, the *n*-th component displacement field *u*_*n*_ at a position *x* from a point source at position ξ can be expressed as


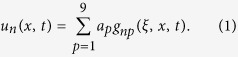


The quantity *g*_np_ is the *n*-th component displacement at station *x* in response to the *p*-th elementary source (*p* = 1–6 for the six elementary moment tensors and *p* = 7–9 for the three orthogonal forces) at position ξ. *a*_*p*_ is the excitation of the *p*-th elementary source. Here, the synthetics are obtained using Green’s functions computed by the propagator matrix approach^26^ for one-dimensional seismic velocity model^19^,^27^. The synthetics are convolved with the source time function and filtered in the same way as the records. The lower corner frequency of 0.025 Hz is chosen such that the spatial scale of the sliding block is small enough compared to the wavelength of the seismic waves to satisfy the block model approximation; while the upper corner frequency of 0.05 Hz is chosen to minimize the influence from local small-scale structures. Sinusoidal- and triangular-shaped source time functions were applied to the SF and MT responses, respectively. Previous study concluded that the modeled SF seismograms are insensitive to the choice of force time function^6^. However, changes in source duration can cause substantial differences in the inverted force magnitudes; thus the estimated force magnitudes should be interpreted with caution. Sinusoidal force time function with a duration of 30 s was used in this study. Due to uncertainties in the event location and origin time and the assumed velocity model, synthetic waveforms do not perfectly align with the records. Therefore, recorded and synthetic waveforms are cross-correlated and the records are shifted with an allowance of ±5 s to maximize the cross-correlation coefficients. The shift is done for each component individually. Finally, the best fit coefficient *a*_*p*_ is determined in a least-squares sense, and the fitness is quantified by both the variance reduction (VR; ref. 28) and the normalized cross-correlation coefficient (CC; ref. 29).For the pure DC component, we adopt an automatic and efficient approach^29^, which is a grid-search scheme based on the genetic algorithm, to determine the fault-plane solutions. Only vertical and radial components were used in the LP waveform modeling for the ISO source component.

### Landquake Force History (LFH)

Seismic waves from a landquake source are generated by time-varying forces acting on the Earth. Following the inversion method developed by Ekström & Stark^8^, we parameterize the force time history of each component (north, east and vertical) using a sequence of 50% overlapping isosceles triangles. We use 7–11 triangles in this study, each with a half-duration of 10 s. The magnitudes of the triangles that define the LFH of each force component are solved by minimizing, in a least-squares sense, the misfit between observed and synthetic seismograms. The time history of each force component is constrained to integrate to zero in order to satisfy the condition that the sliding block must be at rest before and after the landquake event.

## Additional Information

**How to cite this article**: Chao, W.-A. *et al.* Seismology-based early identification of dam-formation landquake events. *Sci. Rep.* **6**, 19259; doi: 10.1038/srep19259 (2016).

## Supplementary Material

Supplementary Information
