# Supplementary material for: Seismology-based early identification of dam-formation landquake events
Source: Sci Rep. 2016 Jan 12;6:19259. doi: 10.1038/srep19259 (PMC4709719; doi:10.1038/srep19259)
Supplement: Supplementary Information [file srep19259-s1.pdf]

Supplementary Information

Seismology-based early identification of dam-formation landquake events

Wei-An Chao[1*], Li Zhao[2], Su-Chin Chen[3], Yih-Min Wu[1,4], Chi-Hsuan Chen[5], and Hsin-Hua Huang[6,7]

1. Department of Geosciences, National Taiwan University, Taipei 10617, Taiwan
2. Institute of Earth Sciences, Academia Sinica, Nankang, Taipei 11529, Taiwan
3. Department of Soil and Water Conservation, National Chung Hsing University, Taichung 40227, Taiwan
4. National Center for Research on Earthquake Engineering, National Applied Research Laboratories, Taipei 10668, Taiwan
5. Central Geological Survey, MOEA, Taipei 23568, Taiwan
6. Department of Geology and Geophysics, University of Utah, Salt Lake City, USA
7. Seismological Laboratory, California Institute of Technology, Pasadena, CA 91125, USA

This PDF file includes:

Table S1: Characteristics of the ten landquakes obtained by waveform inversion in this study.

Figure S1: General source inversion (GSI). Examples of fits at one station between records (black) and synthetic seismograms calculated for different source mechanisms including single force (SF, blue), and full moment tensor (MT) and its deviatoric moment tensor (CLVD+DC), double couple (DC), and isotropic (ISO) components plotted with different gray levels for three event without dam-formation: (a) Huisun, (b) Taimali#1 and (c) Laonong#1, and for two dam-formation landquake events (DFLEs): (d) Taimali and (e) Oso-steelhead. All waveforms are filtered to 0.025-0.05 Hz. The normalized cross-correlation coefficient (CC) and variance reduction (VR) are given at the end of each synthetic trace. The station name, epicentral distance, and station azimuth are given at the top.

Figure S2: Landquake force history inversion (LFH) and landquake dynamics for Event ID Taimali#2. LFH of each component (green: north; blue: east; red: down), time-dependent horizontal force vectors acting on the Earth are shown in the left panel. Color dots in the lower left corner indicate the locations of the center of collapsed-mass along run-out path trajectory. The black dot shows the transition spot from acceleration to deceleration. All color dots correspond to the time progression from 0 to 80 sec in the LFH result. Waveform fits at two stations between records (black) and synthetic (red) seismograms are shown in the right panel. All waveforms are filtered to 0.025-0.05 Hz, and the fitness value is 1.554. The normalized cross-correlation coefficient (CC) and variance reduction (VR) are given at the end of each synthetic trace. The station name,

epicentral distance, and station azimuth are given at the top.

Figure S3: Plots of HF horizontal envelope functions versus epicentral distances for events of Shiaolin (left) and Taimali (right). The slope of the dashed lines indicates propagation speeds of 1 km/s, 2 km/s, 3 km/s and 4 km/s.

Figure S4: Distributions of broadband seismic stations and landquake events. Study area maps for (a) Nine landquake events in Taiwan and (b) Oso-steelhead landquake in Washington, U.S.A. Taiwan map shows the BATS seismic stations (triangles). Cyan triangles indicate seismic stations used in this study. Black dots represent the epicenters of ten landquake events. Maps are created using GMT (Generic Mapping Tools, http://gmt.soest.hawaii.edu/) software.

Figure S5: HF horizontal envelope functions for Station SCZB (top), MASB (middle) and ECLB (bottom).

Table S1.

ID	Station Number	Time of event (UTC)	Long. (°E)	Lat. (°N)	*Fitness*	F_{max} ($\times 10^{10}$ N)	m ($\times 10^{10}$ kg)	D_h (m)	D_v (m)	M_{LQ} ($\times 10^{13}$ kgm)
Huisun	9	2008/09/15 08:59	120.99	24.10	1.314	7.2	6.50	1577	1213	12.94
Shiaolin	9	2009/08/08 22:16	120.67	23.16	1.421	23.3	8.00	2624	1047	25.78
Taimali#1	9	2009/08/08 17:05	120.71	22.50	1.256	2.9	1.80	1330	577	2.61
Taimali#2	2	2009/08/08 18:19	120.71	22.67	1.554	0.8	0.35	1424	507	0.53
Laonong#1	5	2009/08/09 00:34	120.76	23.23	0.979	0.3	0.05	2606	1371	0.15
Laonong	11	2009/08/09 02:52	120.75	23.22	1.148	18.7	7.00	2603	1074	19.72
Taimali#3	12	2009/08/09 09:28	120.80	22.54	1.596	4.4	4.50	1519	844	7.82
Taimali	14	2009/08/09 09:31	120.81	22.55	1.401	61.6	25.00	3000	1358	82.34
Namaxia	9	2009/08/10 04:22	120.77	23.30	0.791	2.5	0.80	2659	1182	2.33
Oso-Steelhead	10	2014/03/22 17:37	-121.85	48.28	1.266	0.8	1.70	881	55	1.50

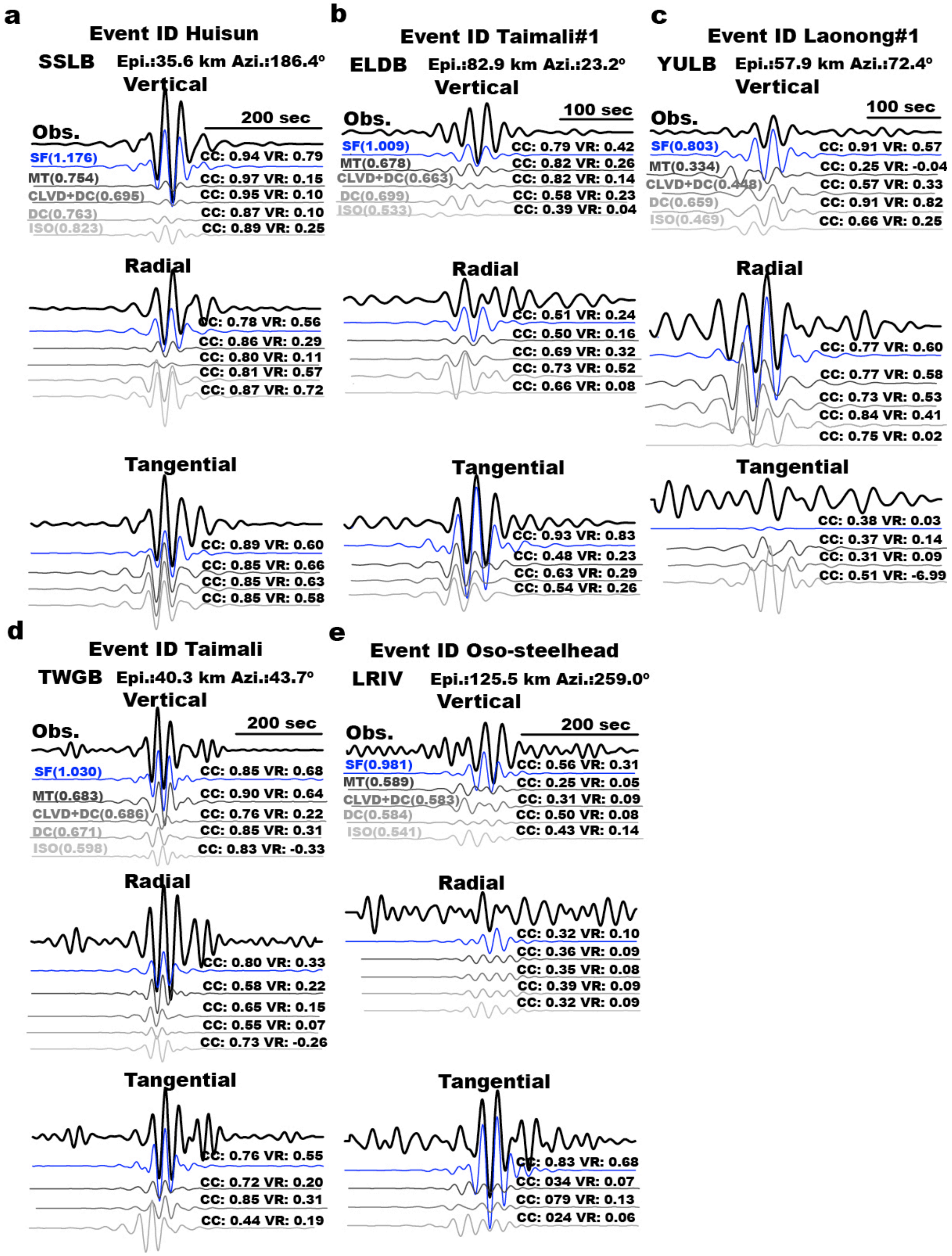
a
Event ID Huisun
SSLB Epi.:35.6 km Azi.:186.4°
Vertical
200 sec
Obs.
SF(1.176)
MT(0.754)
CLVD+DC(0.695)
DC(0.763)
ISO(0.823)
CC: 0.94 VR: 0.79
CC: 0.97 VR: 0.15
CC: 0.95 VR: 0.10
CC: 0.87 VR: 0.10
CC: 0.89 VR: 0.25
Radial
CC: 0.78 VR: 0.56
CC: 0.86 VR: 0.29
CC: 0.80 VR: 0.11
CC: 0.81 VR: 0.57
CC: 0.87 VR: 0.72
Tangential
CC: 0.89 VR: 0.60
CC: 0.85 VR: 0.66
CC: 0.85 VR: 0.63
CC: 0.85 VR: 0.58
b
Event ID Taimali#1
ELDB Epi.:82.9 km Azi.:23.2°
Vertical
100 sec
Obs.
SF(1.009)
MT(0.678)
CLVD+DC(0.663)
DC(0.699)
ISO(0.533)
CC: 0.79 VR: 0.42
CC: 0.82 VR: 0.26
CC: 0.82 VR: 0.14
CC: 0.58 VR: 0.23
CC: 0.39 VR: 0.04
Radial
CC: 0.51 VR: 0.24
CC: 0.50 VR: 0.16
CC: 0.69 VR: 0.32
CC: 0.73 VR: 0.52
CC: 0.66 VR: 0.08
Tangential
CC: 0.93 VR: 0.83
CC: 0.48 VR: 0.23
CC: 0.63 VR: 0.29
CC: 0.54 VR: 0.26
c
Event ID Laonong#1
YULB Epi.:57.9 km Azi.:72.4°
Vertical
100 sec
Obs.
SF(0.803)
MT(0.334)
CLVD+DC(0.448)
DC(0.659)
ISO(0.469)
CC: 0.91 VR: 0.57
CC: 0.25 VR: -0.04
CC: 0.57 VR: 0.33
CC: 0.91 VR: 0.82
CC: 0.66 VR: 0.25
Radial
CC: 0.77 VR: 0.60
CC: 0.77 VR: 0.58
CC: 0.73 VR: 0.53
CC: 0.84 VR: 0.41
CC: 0.75 VR: 0.02
Tangential
CC: 0.38 VR: 0.03
CC: 0.37 VR: 0.14
CC: 0.31 VR: 0.09
CC: 0.51 VR: -6.99
d
Event ID Taimali
TWGB Epi.:40.3 km Azi.:43.7°
Vertical
200 sec
Obs.
SF(1.030)
MT(0.683)
CLVD+DC(0.686)
DC(0.671)
ISO(0.598)
CC: 0.85 VR: 0.68
CC: 0.90 VR: 0.64
CC: 0.76 VR: 0.22
CC: 0.85 VR: 0.31
CC: 0.83 VR: -0.33
Radial
CC: 0.80 VR: 0.33
CC: 0.58 VR: 0.22
CC: 0.65 VR: 0.15
CC: 0.55 VR: 0.07
CC: 0.73 VR: -0.26
Tangential
CC: 0.76 VR: 0.55
CC: 0.72 VR: 0.20
CC: 0.85 VR: 0.31
CC: 0.44 VR: 0.19
e
Event ID Oso-steelhead
LRIV Epi.:125.5 km Azi.:259.0°
Vertical
200 sec
Obs.
SF(0.981)
MT(0.589)
CLVD+DC(0.583)
DC(0.584)
ISO(0.541)
CC: 0.56 VR: 0.31
CC: 0.25 VR: 0.05
CC: 0.31 VR: 0.09
CC: 0.50 VR: 0.08
CC: 0.43 VR: 0.14
Radial
CC: 0.32 VR: 0.10
CC: 0.36 VR: 0.09
CC: 0.35 VR: 0.08
CC: 0.39 VR: 0.09
CC: 0.32 VR: 0.09
Tangential
CC: 0.83 VR: 0.68
CC: 034 VR: 0.07
CC: 079 VR: 0.13
CC: 024 VR: 0.06

Figure S1.

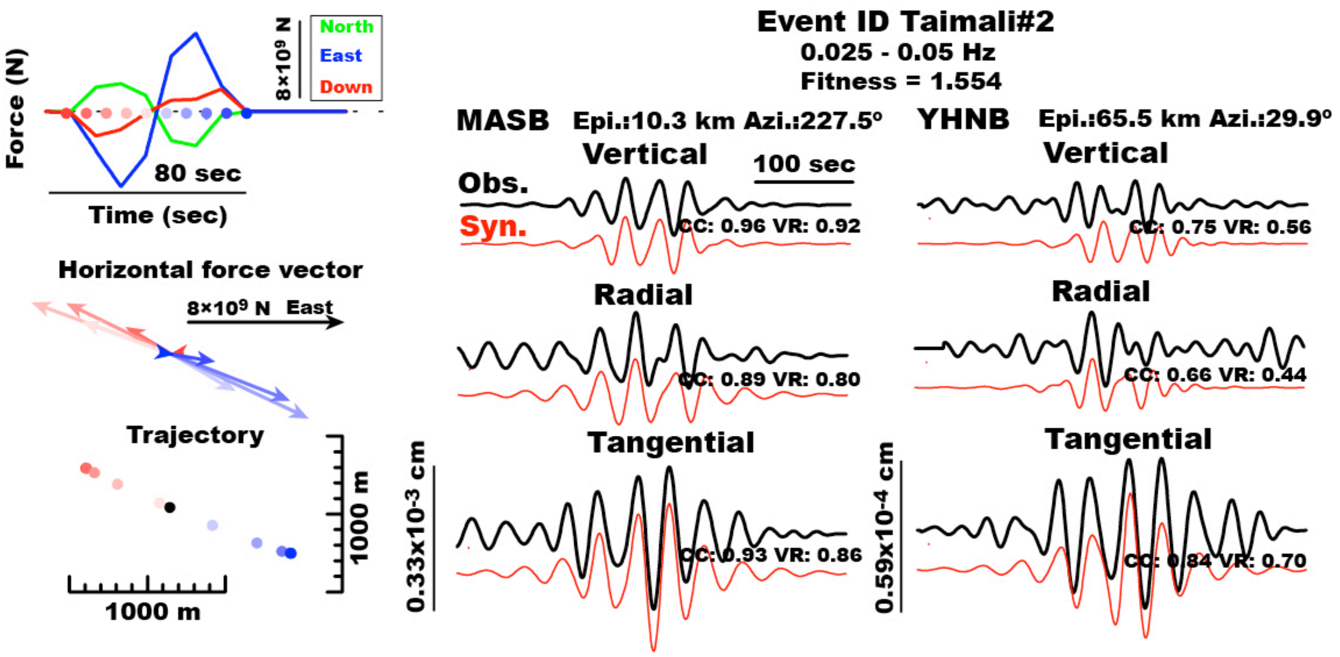
Force (N)
8×10⁹ N
North
East
Down
80 sec
Time (sec)
Horizontal force vector
8×10⁹ N East
Trajectory
1000 m
1000 m
Event ID Taimali#2
0.025 - 0.05 Hz
Fitness = 1.554
MASB Epi.:10.3 km Azi.:227.5°
YHNB Epi.:65.5 km Azi.:29.9°
Vertical
100 sec
Obs.
Syn.
CC: 0.96 VR: 0.92
CC: 0.75 VR: 0.56
Radial
CC: 0.89 VR: 0.80
CC: 0.66 VR: 0.44
Tangential
0.33x10-3 cm
0.59x10-4 cm
CC: 0.93 VR: 0.86
CC: 0.84 VR: 0.70

Figure S2.

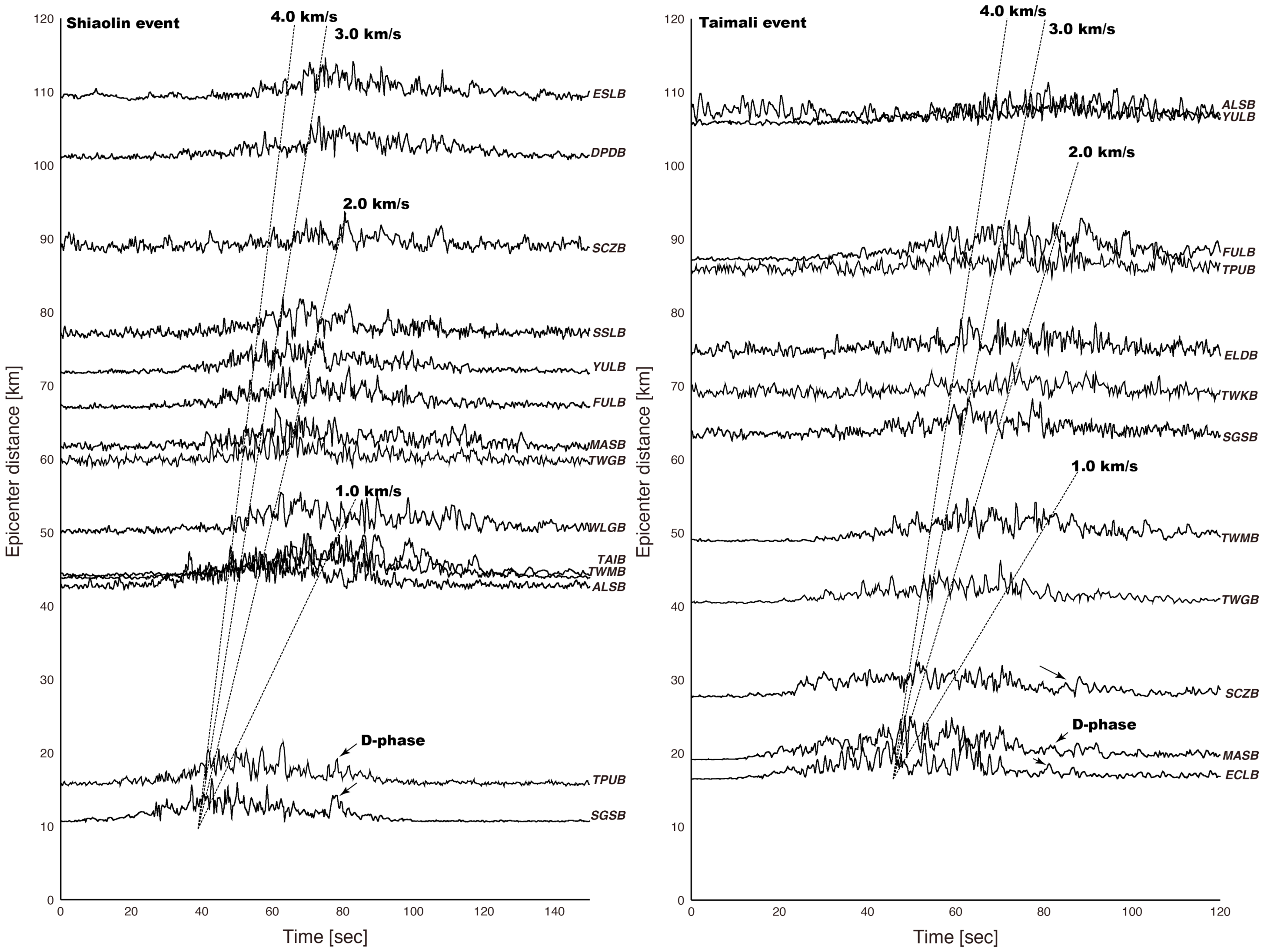

Shiaolin event
4.0 km/s
3.0 km/s
2.0 km/s
1.0 km/s
D-phase
ESLB
DPDB
SCZB
SSLB
YULB
FULB
MASB
TWGB
WLGB
TAIB
TWMB
ALSB
TPUB
SGSB
Epicenter distance [km]
Time [sec]
Taimali event
4.0 km/s
3.0 km/s
2.0 km/s
1.0 km/s
D-phase
ALSB
YULB
FULB
TPUB
ELDB
TWKB
SGSB
TWMB
TWGB
SCZB
MASB
ECLB
Epicenter distance [km]
Time [sec]

Figure S3.

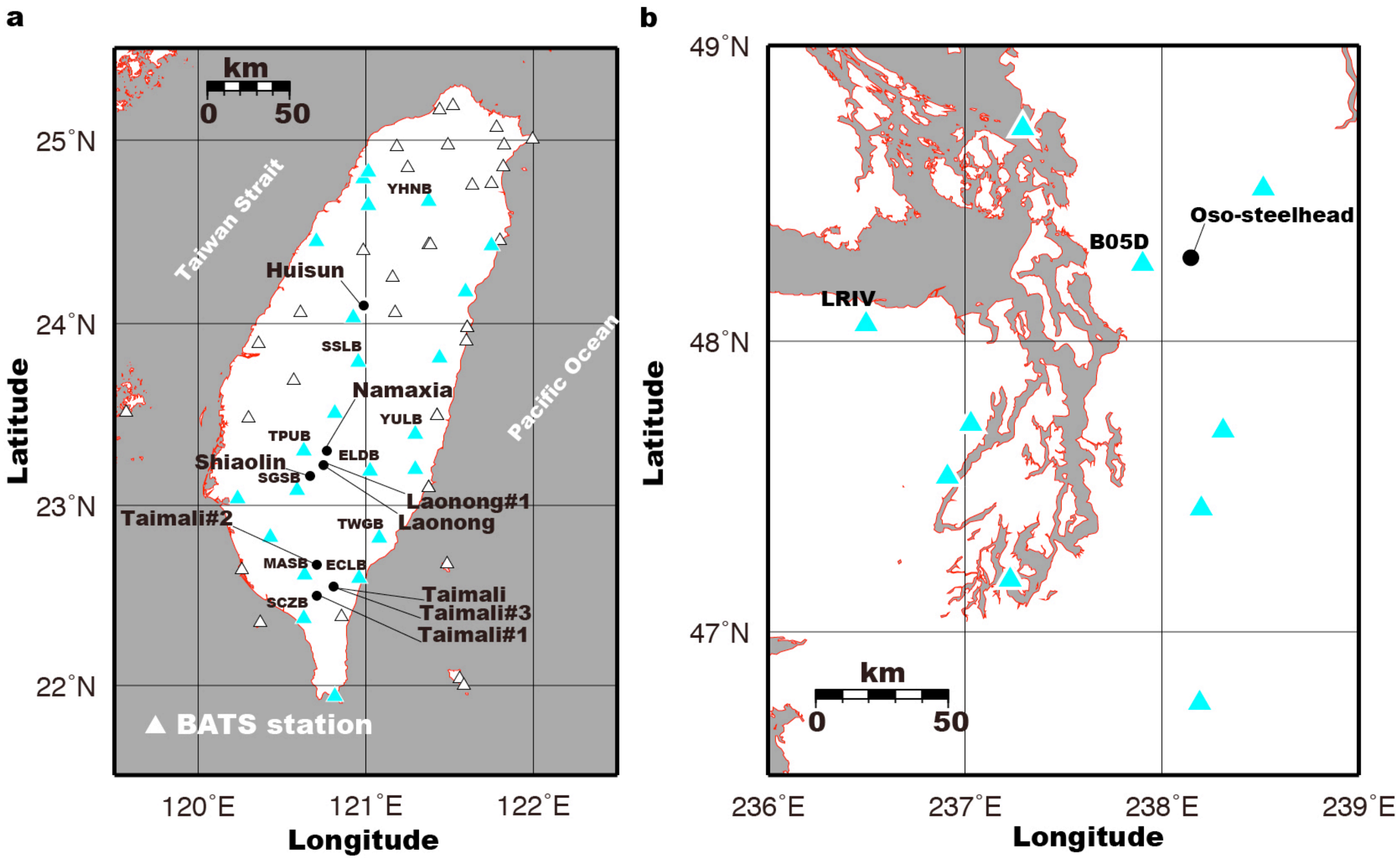
a
b
km
0
50
25°N
24°N
23°N
22°N
120°E
121°E
122°E
Latitude
Longitude
Taiwan Strait
Pacific Ocean
YHNB
Huisun
SSLB
Namaxia
YULB
TPUB
ELDB
Shiaolin
SGSB
Laonong#1
Laonong
Taimali#2
TWGB
MASB
ECLB
SCZB
Taimali
Taimali#3
Taimali#1
BATS station
49°N
48°N
47°N
236°E
237°E
238°E
239°E
Oso-steelhead
B05D
LRIV

Figure S4.

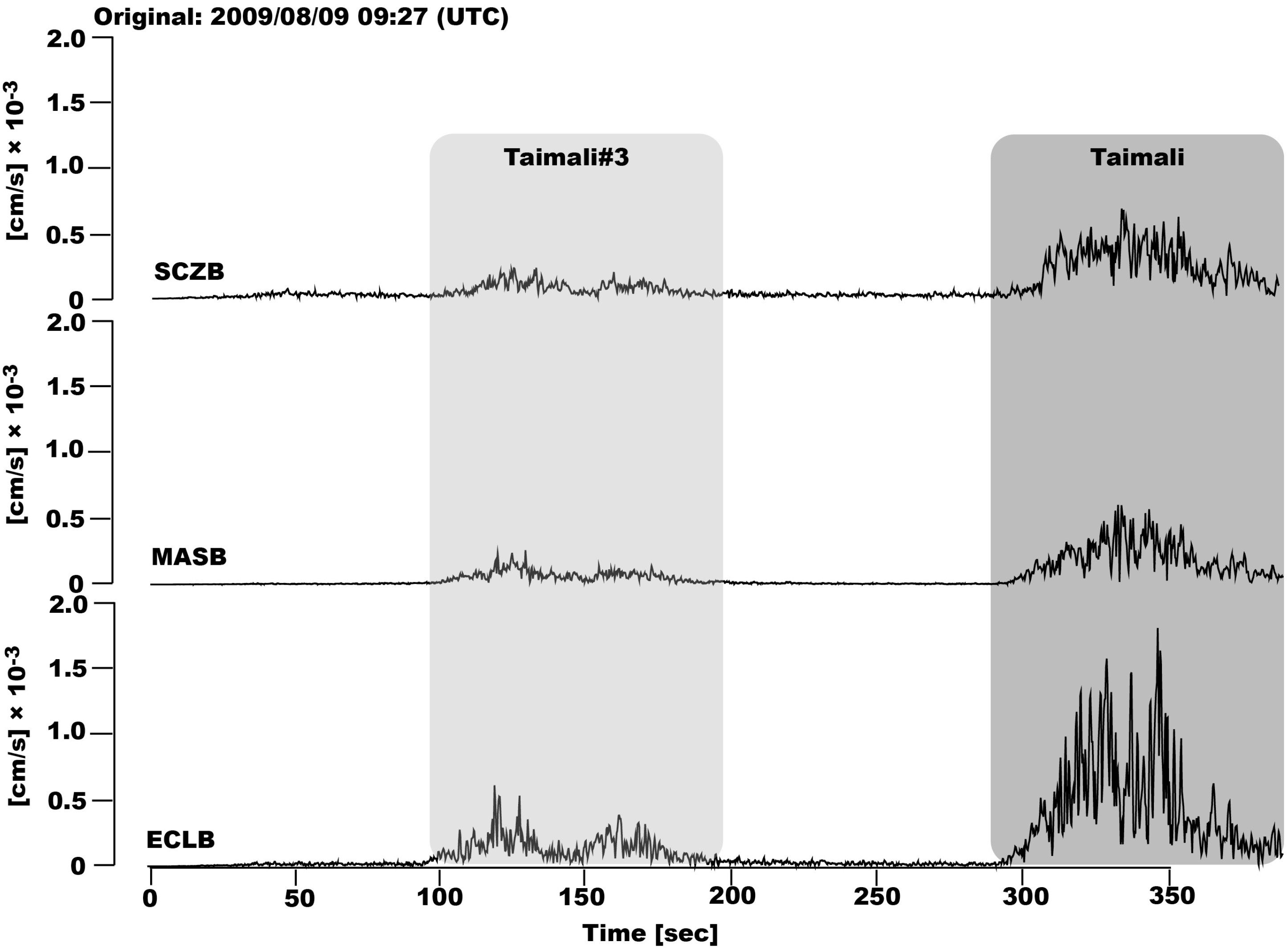
Original: 2009/08/09 09:27 (UTC)
Taimali#3
Taimali
SCZB
MASB
ECLB
[cm/s] × 10-3
2.0
1.5
1.0
0.5
0
0
50
100
150
200
250
300
350
Time [sec]

Figure S5.
